# Heterologous expression of abaecin peptide from *Apis mellifera* in *Pichia pastoris*

**DOI:** 10.1186/s12934-017-0689-6

**Published:** 2017-05-03

**Authors:** Denis Prudencio Luiz, Juliana Franco Almeida, Luiz Ricardo Goulart, Nilson Nicolau-Junior, Carlos Ueira-Vieira

**Affiliations:** 1 Genetics Laboratory, Institute of Genetics and Biochemistry, Federal University of Uberlândia, 1720 Pará, Uberlândia, MG 38400902 Brazil; 2 Nanobiotechnology Laboratory, Institute of Genetics and Biochemistry, Federal University of Uberlândia, 1720 Pará, Uberlândia, MG 38400902 Brazil

**Keywords:** Abaecin, Proline-rich antimicrobial peptide, Heterologous expression, *Pichia pastoris*, *Apis mellifera*

## Abstract

**Background:**

Antimicrobial peptides (AMPs) are the first line of host immune defense against pathogens. Among AMPs from the honeybee *Apis mellifera*, abaecin is a major broad-spectrum antibacterial proline-enriched cationic peptide.

**Results:**

For heterologous expression of abaecin in *Pichia pastoris*, we designed an ORF with HisTag, and the codon usage was optimized. The gene was chemically synthetized and cloned in the pUC57 vector. The new ORF was sub-cloned in the pPIC9 expression vector and transformed into *P. pastoris*. After selection of positive clones, the expression was induced by methanol. The supernatant was analyzed at different times to determine the optimal time for the recombinant peptide expression. As a proof-of-concept, *Escherichia coli* was co-incubated with the recombinant peptide to verify its antimicrobial potential.

**Discussion:**

Briefly, the recombinant Abaecin (rAbaecin) has efficiently decreased *E. coli* growth (*P* < 0.05) through an in vitro assay, and may be considered as a novel therapeutic agent that may complement other conventional antibiotic therapies.

## Background

Insects have cellular and humoral defenses in the innate immunological system. The circulating hemocytes are responsible for the first, as in phagocytosis cases and pathogens nodulation [1, 2] and the production of peptides by the fat bodies related to the second [3].

The antimicrobial peptides (AMPs) are molecules present in the immune system of multinuclear organisms, acting in the defense against invaders such as gram-positive, gram-negative bacteria and fungi [4].

The abaecin peptide, found in *Apis mellifera*, is one of the largest proline-rich antimicrobial peptide, with 34 amino acids containing 10 prolines (29%) and no cysteine residues. The total charge is 4^+^, grouped within positions 12, 13, 27, and 29. Prolines are uniformly distributed through the peptide length, preventing the α-helical conformation [5, 6].

The yeast *Pichia pastoris* is methylotrophic, which means a capacity of using the methanol as its only carbon source. The yeast oxidizes the methanol producing formaldehyde and hydrogen peroxide, using oxygen molecules and alcohol oxidase enzymes. The yeast produces large quantity of this enzyme, due to its low affinity to oxygen, being regulated by the alcohol oxidase 1 promoter (AOX1). The AOX1 promoter within the vector, induced by methanol, leads to expression of the gene of interest at high levels. The protein expression using the pPIC9 vector occurs in an extracellular manner, decreasing the steps between expression and obtaining the peptide of interest [7, 8].

Antimicrobial peptides were isolated from many kind of organism like vertebrates and invertebrates animals, plants, bacteria, fungi, viruses and artificially synthesized in laboratory for experiments [9]. However, our aim was to construct a synthetic gene to express the abaecin peptide from *A. mellifera* in *P. pastoris*. Our purpose was to optimize the expression of this recombinant antimicrobial peptide with biological effects, leading to a possible new drug development.

## Methods

### Gene design and synthesis

The recombinant peptide coding sequence was designed based on the deposited sequence in the National Center for Biotechnology Information (NCBI) NP_001011617.1, of *A. mellifera*’s abaecin peptide. Some nucleotides sequences were added to facilitate cloning and optimization of this protein expression in yeast *P. pastoris.* Two restriction sites were added: in the 5′ position for EcoRI enzyme, and another one in 3′ position for NotI enzyme. It was also added hexahistidine-tag (Histag) for expression confirmation process, and a stop codon was included as well. The peptide signal of the entire abaecin sequence was not used.

The sequence was optimized with the following parameters: length: 129, GC%: 45.08, GAATTC-TACGTTCCATTGCCTAACGTTCCACAACCTGGTAGAAGACCATTTCCTACTTTCCCAGGTCAAGGACCTTTTAACCCTAAGATTAAATGGCCTCAGGGATATCGTCGACATCACCATCACCATCACTAA-GCGGCCGC, therefore, the new abaecin sequence was deduced: YVPLPNVPQPGRRPFPTFPGQGPFNPKIKWPQGYHHHHHH. Then, to optimize the sequence for *P. pastoris*, the codon adaptation index (CAI) was used. It measures the extension level of differential usage of codons in highly expressed genes [10]. The measurements in this technique use the OptimumGeneTM (GenScript^®^ Corporation) software.

After analysis and deduction of the optimized DNA sequence, the chemical synthesis of the minigene was performed, cloned in pUC57, and sequenced for confirmation (GenScript^®^ Corporation).

### Sub-cloning of the synthetic gene in the expression vector pPIC9

The insert was removed from vector pUC57 using the NotI and EcoRI enzymes. The vector pPic9 was also digested with the same enzymes, quantified and dephosphorylated with SAP enzyme. It was used 16.7 ng of the insert in the ligation reaction with 50 ng of pPIC9 vector and 1U of the DNA ligase I (Invitrogen) at 14 °C overnight. The product of this reaction was named pPic9abacin, confirmed by electrophorese at 0.8% of agarose. The *Escherichia coli* Top10 bacteria were transformed with the pPic9abaecin by electroporation in 0.2 cm cuvette with lysogeny broth (LB), following the parameters: 2.5 kV, 200 Ω and 25 µF, electroporated in Bio-Rad Gene Pulser (Bio-Rad). These clones were cultivated in LB medium containing 50 µg/mL of Ampicillin. The plasmid extraction was performed using QIAprep^®^ (Qiagen^®^), following the kit protocol.

### Transformation and electroporation of *Pichia*

The recombinant plasmid was linearized with SacI enzyme (FastDigest, Fermentas) 1 μL/μg (enzyme/DNA), purified with phenol/chloroform/125:24:1 and used to transform the strain GS115 *P. pastoris* yeast. The GS115 strain was cultivated in 50 mL of yeast extract peptone dextrose (YPD, 1% yeast extract, 2% peptone and 2% dextrose) at 30 °C overnight, then inoculated 0.5 mL of this culture in 500 mL of fresh YPD at 30 °C overnight until OD_600_ = 1.3–1.5. The cells were centrifuged at 1500×*g* for 5 min at 4 °C and resuspended, for each of the followed steps: 500 and 250 mL sterilized water, then in 20 and 1 mL of sorbitol 1 M, all processes realized in ice-cold. The transformation system of 10 µg of linearized DNA in 10 μL TE Buffer (10 mM Tris–HCl, 1 mM EDTA, pH 8.0) and 80 µL of competent *P. pastoris* cells was incubated in ice-cold electroporation cuvette of 0.2 cm for 5 min in ice. The electroporation followed the parameters of 1.5 kV, 200 Ω and 25 µF, cells were immediately incubated with 1 mL of 1 M sorbitol and spread 200 μL of aliquots on RDB plates (regeneration dextrose base, 9.3:1 sorbitol/agar, w/w) without histidine. The plates were incubated at 30 °C until colonies appear [8]. The transformation was confirmed by PCR and electrophorese in agarose gel 1.5% of the clones.

### Expression of the recombinant peptide

A recombinant colony was inoculated in buffered minimal glycerol (BMG) medium containing 100 mM potassium phosphate, pH 6.0, 1.34% (w/v) of yeast nitrogen base (YNB), 4 × 10^−5^% (w/v) of biotin, and 1% glycerol, for two days at 28 °C. Then, the cells were centrifuged and re-suspended in buffered minimal methanol (BMM) medium, composed of 100 mM of potassium phosphate, pH 6.0, 1.34% (w/v) YNB, 4 × 10^−5^% (w/v) biotin, and methanol 0.5%, in order to avoid the action of proteases [11]. at the optical density OD_600_ = 1.0, under the temperature of 28 °C, at 250 rpm.

To better verify the induction time of expression rates, supernatants were collected at times 0, 6, 12, 24, 36, 48, 60, 72 and 96 (h) with the addition of 0.5% (w/v) of methanol at times 0, 24, 48 and 72 (h). The expression confirmation was analyzed by Tricine-SDS-PAGE electrophoresis [12], and silver-stained.

### Growth inhibition test

The inhibition test was done in 96-well microtiter plate containing, in the first row, 200 µL of LB, as negative control. A mix containing 200 µL of LB with *E. coli* DH5α, in an OD_595_ = 0.3, was added in the second row as first positive control (only *E. coli*). In the third, fourth and fifth rows was added 200 µL of LB with *E. coli* and new lyophilized BMM medium in quantities of 1, 10 and 25 µg, respectively, control for supernatant without recombinant peptide (named BMM). In the sixth, seventh and eighth rows were added 200 µL of LB with *E. coli* and BMM medium with abaecin in quantities of 1, 10 and 25 µg, respectively. The test was run in quadruplicated and the average of OD values from the negative control were subtracted from all wells.

### Abaecin peptide modeling

The abaecin peptide was modeled by ab initio method, which is used when there is little or no initial information about the molecule structure in the data banks. The Rosetta 3.5 software [13], specifically the protocol of the AbinitioRelax software [14–19] was used to this goal. Approximately 2000 models were generated, one being selected after evaluation and validation using the dDFIRE [20]. The dDFIRE generates a score based on the free energy of the structure, therefore we select, among the thousands of models, one who has minor free energy. The peptide was visualized using the Chimera 1.1 software [21], where views of surface of the Coulombic electrostatic potential and amino acids hydrophobicity were generated.

### Statistical analysis

The data obtained was analyzed using the Prism 4.0 software (GraphPad, San Diego, CA). After verification of data distribution, it was used analysis of variance for repeated variables, and Bonferroni tests to compare the obtained p value (*P* < 0.05).

## Results

### Gene design and synthesis and codon optimization

After the deduction and optimization of the new gene sequence, the abaecin of *A. mellifera* and the modified abaecin sequences were aligned by Clustal W software to check the non-modified regions and the optimized codons. The chemically synthesized gene of abaecin, with the size of 143 bp, was cloned into pUC57 clone vector.

### Expression of recombinant peptide

**Fig. 1:**
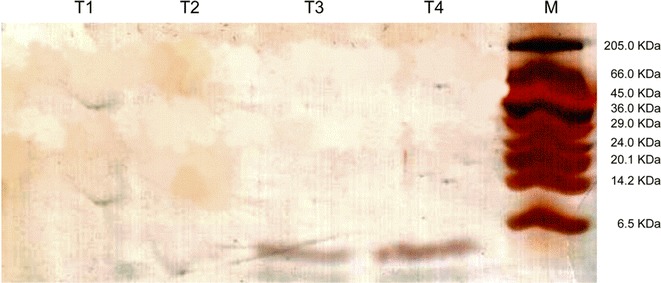
Electrophoresis Tricine-SDS-PAGE, silver-stained. Expression time: T1 (24 h), T2 (48 h), T3 (72 h), T4 (96 h) with addition of 0.5% of methanol at all times. It shows the production of the peptide abaecin, of approximately 5.2 kDa, migrating lower than the last band of the marker (M) of 6.5 kDa. The beginning of the peptide’s expression happened at the 72 h of incubation under 30 °C

The colonies of *P. pastoris* transformed with pPic9abaecin were grown in BMG medium for development and then in BMM for expression. Supernatants were collected from the expression medium in different times (Fig. 1). At times T3 (72 h) and T4 (96 h), it is possible to visualize the 5.2 kDa band showing that the selected colony produced the isolated peptide, not excluding the possibility of also containing peptides in the truncated form.

### Inhibition growth test

Due to non-purification of the peptide in the present study, we adapted the antibiogram assay using the crude expression supernatant. After the confirmation of the production of recombinant peptide, the supernatant containing peptide was lyophilized and tested in *E. coli* culture in 96-well microtiter plate for antimicrobial activity confirmation.

**Fig. 2:**
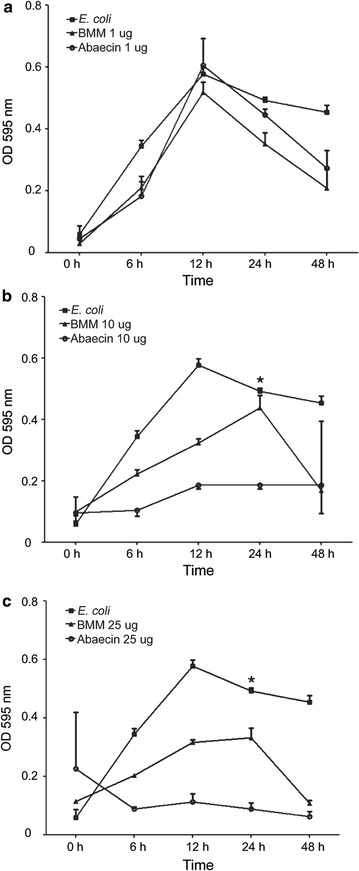
Graphics of optical density (OD) measurements by time in the analyzed wells. *Square E. coli* (first positive control); *triangle* BMM (LB with *E. coli* and new lyophilized BMM medium without recombinant peptide); and *circle* abaecin (LB with *E. coli* and BMM medium with recombinant abaecin). The graphics **a**–**c** show three different quantities of lyophilized BMM medium with and without abaecin, 1, 10 and 25 µg, respectively. *Significant difference compared to BMM sample (*P* < 0.01, Bonferroni test) and abaecin (*P* < 0.001, Bonferroni test) at time 24 h

The test showed that the quantities of 10 and 25 µg of lyophilized medium, containing the recombinant peptide, was sufficient to significantly inhibit the *E. coli* growth after 24 h treatment (Fig. 2b, c). There was no interference in the abaecin peptide inhibition by the lyophilized BMM medium. The quantity of 1 µg did not inhibit bacterial growth (Fig. 2a).

The statistical test showed significant difference in rAbaecin activity in relation to controls (*E. coli* and BMM only). It presented antibacterial activity in concentrations of 10 and 25 µg, respectively, after time 12, accentuating after 24 h.

### Abaecin structure

**Fig. 3:**
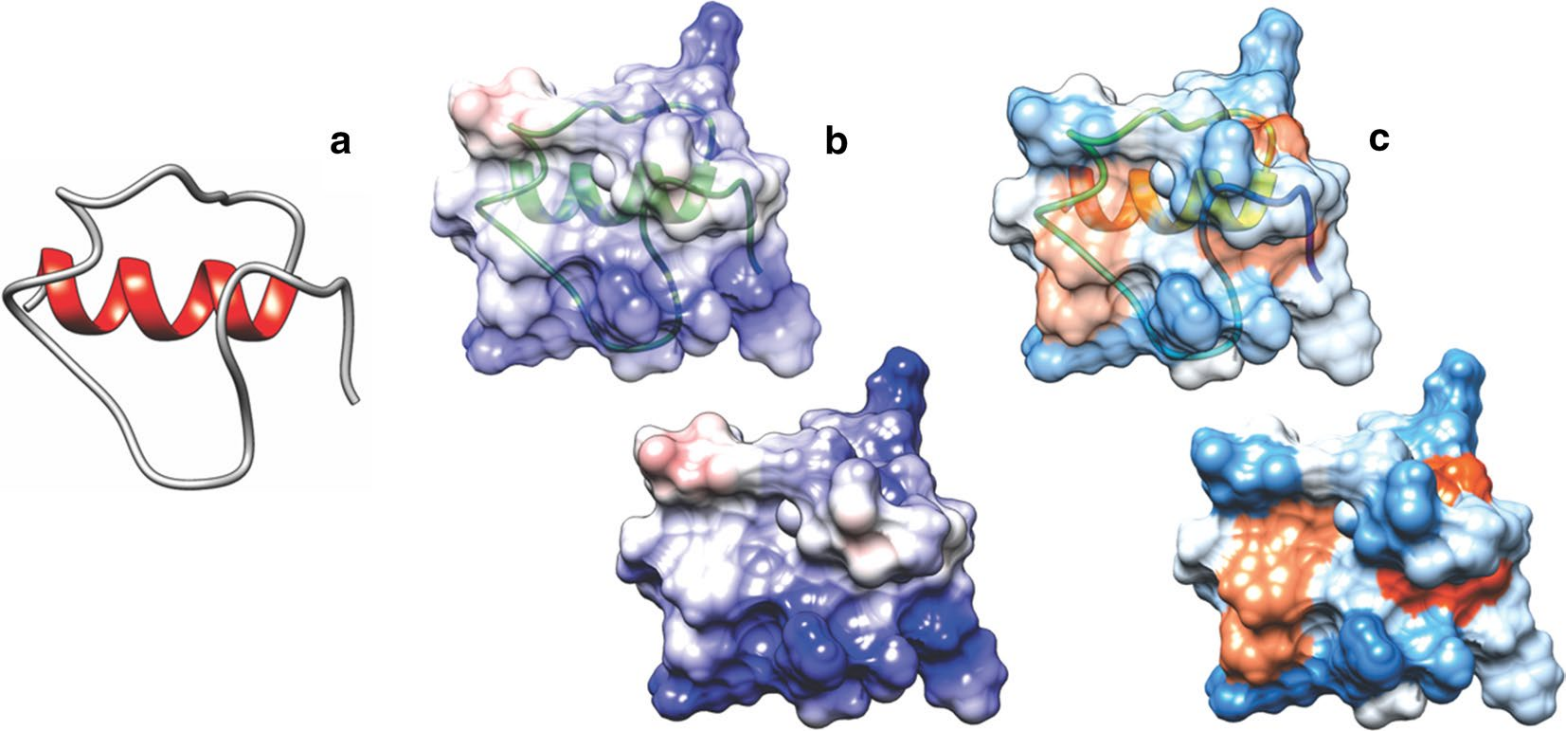
Structural analysis of the heterologous peptide. In **a** structure abaecin with α-helix in red, in **b** analysis of electrostatic surface: positive charges in blue and negative charges in red, and **c** hydrophobicity surface analysis, warmer colors indicate hydrophobic regions

The peptide sequence optimized abaecin (43 aas + histag) showed a α-helix structure in C-terminal portion composed by (KWPQGYHHHHHH) residues in red on structural analyses. The views of surface of the Coulombic electrostatic potential and amino acids hydrophobicity showed in (Fig. 3). This conformation suggests an interaction with the bacterial membrane.

## Discussion

The antimicrobial effect of peptides, and their production, has been studied in the immunological system of animals, such as bees. A study on the bee specie *Bombus pascuorum* identified four types of antimicrobial peptides: defensin, hymenoptaecin, apidaecin and abaecin. These peptides were directly extracted from the animal’s hemolymph, which were purified and tested in bacterial culture, presenting antimicrobial activity [22].

In wasps from the specie *Pteromalus puparum* an abaecin cDNA was identified. The protein was chemically synthetized and tested in *E. coli* culture using this cDNA sequence, showing inhibitory activity, and no hemolytic activity was shown [23].

Previous DNA recombination methods were heavily used to high levels of peptide expression [24, 25]. For a long time, the system of proteins expression by *E. coli* was used due to its easy maintenance and fast culture growth [26]. It was not possible to use *E. coli* as an expression system because the recombining product is toxic to the bacteria. Because of that, the *P. pastoris* expression system was used.

The *P. pastoris* yeast, being a eukaryote, has advantages in the protein expression and processing, being able to make post-translational modifications. Another great advantage is the fact that, it is easier to manipulate when compared to both *E. coli* and *Saccharomyces cerevisiae* expression. The methodology used is also faster and cheaper compared to other expression systems like baculovirus or other culture tissues, allowing a high expression index [8, 27, 28].

The use of *P. pastoris* yeast has been used as an important protein expression method. Studies of antimicrobial peptides based on shrimp [29], butterfly, frog [30], *Drosophila melanogaster*, spine soldier bug [31] and human [32] genes have proven the effectiveness of this system for the production of these peptides without affecting their inhibitory activity. Here we describe a production of recombinant abaecin (from *A. mellifera*) in *P. pastoris* host.

The chemical synthesis of the abaecin gene from *A. mellifera*, to express the peptide in *P. pastoris* yeast, allowed the production of the antimicrobial peptide. This system, which has shown capacity of a fast production and easy maintenance, can be used to deliver the expected results. The use of pPIC9 vector of expression allowed the peptide to be expressed in an extracellular way, reducing the number of steps in the manipulation methodology. By using the expression optimization, it was possible to estimate the necessary time for the production.

In order to the abaecin peptide be tested in *E. coli* samples, the optimum incubation period was 24 h. After this time the OD of the analyzed samples decreases, supposedly because bacterial growth reaches a high concentration, causing bacterial death due to the lack of supply and low environmental conditions in the culture mediums [33]. The treated samples with the abaecin in the lyophilized BMM medium peptide at 10 µg, showed an antibacterial activity after 12 h keeping an OD lower than 0.2. On the other hand, the sample treated with 25 µg showed antibacterial activity profile between 6 and 12 h with an OD close to 0.1 (Fig. 2).

The antibacterial activity depend on the hydrophobicity and the structural ability to assume an amphipathic helical conformation [34, 35]. Other patterns affect the potency and spectrum of the AMPs α-helix, such as sequence, charge and amphipathicity. These features are interrelated and they are the key for the development of more potent AMPs with a straight action [36, 37].

Antimicrobial peptides with more than 20 aas proline rich, like abaecin, showed effectiveness against gram-positive bacteria and fungi [38–40]. In another study, the chemically synthetized abaecin peptide, based on *B. pascuorum*, didn’t show any effect without the aid of another peptide. When combined with other peptide, it exhibited action against *E. coli* growth [41]. Our heterologous abaecin, expressed in the *P. pastoris* system, showed antibacterial activity without the use of another AMP.

The charge of the proline-rich AMPs peptides (verified by in silico modeling) may provide the crossing of the membrane cell in non-lytic manner, interacting with proteins from the cytoplasm of the bacterium hampering protein synthesis [42]. This process shortens the quantity of the total bacteria in the samples, showed by the decreasing levels of the OD in the reading 595 nm in the test (Fig. 2).

It was shown that the methodology used facilitated the results of the proposed study. The chemical synthesis based in the sequence of the abaecin gene of *A. mellifera*, and optimized to *P. pastoris* (pPic9abaecin) proved to be efficient inside the expression system. The pPIC9 vector was of great importance to simplify the steps of the process, enabling it to obtain the peptide abaecin in the supernatant expression medium. The rAbaecin expressed in a heterologous manner at time 72 h, showing that there is an inhibition variation of microbial growth of *E. coli* DH5α bacteria within both time 6, 12 and 24 h.

Further studies are needed to the large-scale production and purification of this compound, in order to produce an antibiotic with such elements.

This results also shows that the heterologous abaecin peptide has antimicrobial activity against *E. coli*, and have biotechnological potential for the production of new antimicrobial drug, which acts against bacteria resistance to current drugs.

## Abbreviations

AMP: antimicrobial peptides
AOX 1: alcohol oxidase 1 promoter
pPic9abacin: pPIC9 vector-insert
rAbaecin: recombinant abaecin
YPD: yeast extract peptone dextrose
RDB: regeneration dextrose base
BMG: buffered minimal glycerol
YNB: yeast nitrogen base
BMM: buffered minimal methanol
